# Motion‐corrected MRI with DISORDER: Distributed and incoherent sample orders for reconstruction deblurring using encoding redundancy

**DOI:** 10.1002/mrm.28157

**Published:** 2020-01-03

**Authors:** Lucilio Cordero‐Grande, Giulio Ferrazzi, Rui Pedro A. G. Teixeira, Jonathan O'Muircheartaigh, Anthony N. Price, Joseph V. Hajnal

**Affiliations:** ^1^ Centre for the Developing Brain, School of Biomedical Engineering and Imaging Sciences King's College London London UK; ^2^ Biomedical Engineering Department, School of Biomedical Engineering and Imaging Sciences King's College London London UK; ^3^ Department of Forensic and Neurodevelopmental Sciences, Institute of Psychiatry, Psychology and Neuroscience King's College London London UK

**Keywords:** distributed and incoherent sampling, image reconstruction, magnetic resonance, motion correction, parallel imaging

## Abstract

**Purpose:**

To enable rigid body motion‐tolerant parallel volumetric magnetic resonance imaging by retrospective head motion correction on a variety of spatiotemporal scales and imaging sequences.

**Theory and methods:**

Tolerance against rigid body motion is based on distributed and incoherent sampling orders for boosting a joint retrospective motion estimation and reconstruction framework. Motion resilience stems from the encoding redundancy in the data, as generally provided by the coil array. Hence, it does not require external sensors, navigators or training data, so the methodology is readily applicable to sequences using 3D encodings.

**Results:**

Simulations are performed showing full inter‐shot corrections for usual levels of in vivo motion, large number of shots, standard levels of noise and moderate acceleration factors. Feasibility of inter‐ and intra‐shot corrections is shown under controlled motion in vivo. Practical efficacy is illustrated by high‐quality results in most corrupted of 208 volumes from a series of 26 clinical pediatric examinations collected using standard protocols.

**Conclusions:**

The proposed framework addresses the rigid motion problem in volumetric anatomical brain scans with sufficient encoding redundancy which has enabled reliable pediatric examinations without sedation.

## 1 INTRODUCTION

Tolerance against motion is desirable in magnetic resonance imaging (MRI). This includes brain MRI, where significant motion‐induced image degradation prevalence has been documented^1^ and high‐resolution imaging quality may be compromised by head motion.^2^ Rigid‐body MRI motion correction^3^, ^4^ can be tackled via prospective or retrospective techniques. Prospective techniques^5^ compare advantageously in terms of spin‐history, dephasing confounders, or k‐space density guarantees. Particularly, optical tracking systems have been proposed for head motion estimation, with corrections showing impressive accuracy and latency.^6^ However, prospective methods require additional hardware and/or scanner modifications, often involving intrusive markers attached to the subject. In addition, satisfactory corrections may not always be possible due to unpredictability or complexity of motion, or maximized sampling efficiency requirements. Retrospective techniques may facilitate scanning or improve prospective results,^7^ particularly for 3D encodings, where spin‐history is less of a problem, and when using nonlinear reconstruction paradigms^8^ to deal with nonhomogeneous sampling density after motion.

Motion compensation is strongly dependent on motion estimation from the measured information. Some methods have proposed the use of navigators, where surrogate motion‐sensitive information is interleaved with the main acquisition and correction is applied either prospective or retrospectively.^9^, ^10^, ^11^ Due to variability in time requirements for different MRI sequences, application of a given navigator is usually limited to a specific sequence type. Furthermore, particular care has to be taken to prevent spin‐history or saturation and, sometimes, scanning efficiency may be compromised. Alternatively, sequences can be constructed with relative resilience to motion or, similarly, sampling schemes can be designed to function as implicit navigators. This is the case for spiral and radial trajectories,^12^, ^13^, ^14^ where temporally distributed low‐resolution information is used for motion estimation, with retrospective corrections usually grounded on an intermediate reconstruction of fully formed images for each motion state, often involving nonlinear methods. Finally, other approaches have explored the redundancy of the information sensed by parallel MRI to detect and discard localized inconsistencies in k‐space measurements,^15^ usually requiring prior image models to limit noise amplification and improve inconsistency detection.^16^

Building on models of MRI acquisition in the presence of motion,^12^, ^17^ some methods have proposed formulations for motion estimation from the k‐space that do not require navigators.^18^, ^19^, ^20^ Our previous work^20^ introduced a data‐driven reconstruction method for retrospective multi‐shot rigid‐body motion correction or *aligned reconstruction* taking advantage of the encoding redundancy in the measured data. Performed simulations showed that the ability to solve the aligned reconstruction problem is strongly sensitive to the k‐space encoding order, which suggested that opportunities exist to maximize the sensitivity to motion by appropriate sampling order designs. Consequently, in this paper we introduce the Distributed and Incoherent Sample Orders for Reconstruction Deblurring using Encoding Redundancy (DISORDER) framework as a flexible way to correct for head motion on a variety of spatiotemporal scales and imaging contrasts by optimizing the *sample orders* for k‐space coverage. In addition, we propose some technical refinements to the aligned reconstruction formulation and extend the simulation domain. The technique is implemented on a 3 T scanner and tested on controlled motion scans and pediatric examinations including magnetization‐prepared rapid acquisition gradient echo (MP‐RAGE), fast spin echo (FSE), fluid attenuated inversion recovery (FLAIR), spoiled gradient echo (SPGR), and balanced steady‐state free precession (bSSFP) sequences. A matlab implementation to reproduce the experiments is made available at https://github.com/mriphysics/DISORDER/releases/tag/1.1.0.

## 2 THEORY

### 2.1 Aligned reconstruction

Assuming whitened measurement noise,^21^ the aligned reconstruction for parallel volumetric imaging can be formulated as:

(1) $$\left(\hat{x},\;\hat{\theta }\right)=\underset{x,\;\theta }{\text{argmin}}r_{x,\;\theta }\;=\;\underset{x,\;\theta }{\text{argmin}}{\left\|AFST_{\theta }x-y\right\|}_{2}^{2},$$

where **x** is the image to be reconstructed, ***θ*** are the motion parameters, *r* is the loss function, **y** is the measured k‐space data, **T** is a set of rigid motion transformations, **S** are the coil sensitivities, F is the discrete Fourier transform (DFT), and **A** is a sampling mask. We are interested in reconstructing a 3D image of size $V\;=\;V_{1}V_{2}V_{3}$ with $V_{d}$ the number of voxels along dimension *d* from $N\;=\;C\sum_{m\;=\;1}^{M}E_{m}$ *C*‐element coil array samples of a discretized k‐space grid of size *K*. $E_{m}$ denotes the number of samples within *segment m* and *M* is the number of segments in the sequence, with each segment associated to a specific motion state. Detailed information about the terms in Equation 1 can be found in.^20^ Here we provide a brief description of their structure:

1. **y** is a *N* × 1 vector.
2. **A** is a *N* × *KMC* block matrix comprising submatrices of size $E_{m}\;\times \;K$ whose entries take the value 1 if the sample *e* of the segment *m* corresponds to the k‐space location indexed by *k* and 0 otherwise.
3. F is a *KMC* × *VMC* block diagonal matrix comprising submatrices of size *K* × *V* representing 3D DFT's with applied k‐space sampling.
4. **S** is a *VMC* × *VM* block matrix comprising diagonal submatrices of size *V* × *V* whose diagonal elements correspond to the spatial sensitivity of the coil *c*.
5. **T** is a *VM* × *V* block matrix comprising unitary^22^ submatrices of size *V* × *V* corresponding to the 3D rigid transformation modeling the motion state *m* by three translations and three Euler rotation angles codified in the parameter vector $\theta_{m}$.
6. **x** is a *V* × 1 vector.

Equation 1 is a separable nonlinear least squares problem.^23^, ^24^ We confront it by iteratively addressing the subproblems:

(2) $$\begin{array}{cc} {\hat{x}}^{\left(i+1\right)}\;=\; & \underset{x}{\text{argmin}}{\left\|AFST_{{\hat{\theta }}^{\left(i\right)}}x-y\right\|}_{2}^{2}\\ {\hat{\theta }}^{\left(i+1\right)}\;=\; & \underset{\theta }{\text{argmin}}{\left\|AFST_{\theta }{\hat{x}}^{\left(i+1\right)}-y\right\|}_{2}^{2}. \end{array}$$

The first subproblem, reconstructing the image **x** in the presence of rigid motion,^17^ can be solved by conjugate gradient (CG).^21^ As for the second, the solution must null the gradient of the objective function against the motion parameters,^20^ which is tackled by a Levenberg‐Marquardt (LM) algorithm using a simplified Jacobian.^25^ A natural initialization is a zero‐motion condition ${\hat{\theta }}^{\left(0\right)}\;=\;0$, so the first step corresponds to a standard sensitivity encoding (SENSE) reconstruction. Further in this paper, we describe how to temporally arrange the k‐space samples into segments to improve the aligned reconstruction convergence.

### 2.2 DISORDER sampling

**Figure 1:**
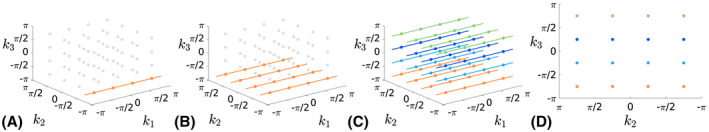
Sketch of volumetric sampling for an exemplary *K* = 4 × 4 × 4 space (in radians). A, Measured samples after acquiring the first readout. B, Measured samples after acquiring the first segment. C, Measured samples after whole sequence acquisition with color coding used to differentiate each segment. D, View of the $k_{2}$‐$k_{3}$ PE plane

We focus on Cartesian 3D k‐space grids with uniform sampling as sketched in Figure 1. Figure 1A shows $K_{1}\;=\;4$ collected samples after the first readout or profile in the $k_{1}$ direction. Figure 1B shows the first segment, in this example corresponding to the full acquisition of the $k_{1}$‐$k_{2}$ plane. Figure 1C shows that segments can be used to define an ordered partition of the $k_{1}$‐$k_{2}$‐$k_{3}$ grid. Due to short duration, we assume negligible motion during the readout and focus on the phase encode (PE) plane in Figure 1D. We define $E_{m}^{\mathrm{PE}}$ as the number of profiles per segment, so $E_{m}^{\mathrm{PE}}\;=\;E_{m}/K_{1}$, and hereinafter we adopt the replacement $E_{m}\leftarrow E_{m}^{\mathrm{PE}}$.

**Figure 2:**
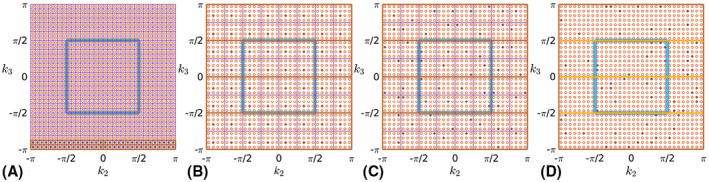
Example of segments for different encoding orders to cover a $P\;=\;K_{2}K_{3}$ PE plane (in radians) with $K_{2}\;=\;K_{3}\;=\;32$ using an acquisition partitioned into *M* = 16 segments with equal number of profiles per segment $E_{s}\;=\;E\;=\;P/M\;=\;64$. Full set of profiles to cover the PE plane as red circles, profiles within a segment filled in blue, underlying tilings ($U_{2}\;=\;U_{3}\;=\;4$) in purple, samples at half the spatial resolution enclosed in cyan, and areas covered by four intra‐segment temporal subdivisions of considered segment enclosed in yellow. A, Sequential; B, Checkered; C, Random‐checkered; and D, Random traversals

By modifying the PE gradients before each readout, it is possible to design different encoding or view orders. These can be defined as a temporally ordered set of profiles $p\in P\;=\;\{\left(k_{2}^{1,1},\;k_{3}^{1,1}\right),\;\;\ldots ,\;\;\left(k_{2}^{1,E_{1}},\;k_{3}^{1,E_{1}}\right),\;\;\ldots ,\;\;\left(k_{2}^{M,\;E_{M}},\;k_{3}^{M,E_{M}}\right)\}$ with cardinality $P\;=\;|P|\;=\;\sum_{m}E_{m}$. Figure 2A shows the first segment of a commonly used Sequential ordering scheme. In this case, due to the partition definition, a segment includes two consecutive $k_{3}$ planes. Figure 2B, introduces the Checkered traversal. First, a rectangular tiling of the PE plane is built using tiles of size $U_{2}\;\times \;U_{3}$ such that $U_{2}U_{3}\;=\;M$. Second, a spectral lexicographic order for the profiles within a tile $K_{U}$ is defined by $K_{U}\to M_{U}\;=\;\left\{1,\;\ldots ,\;M\right\}$, which can be extended to different tiles by translation. Third, interleaved segments are defined such that the same profile $m_{u}\in M_{U}$ is used ∀e∈E={1,…,E}, with E a temporally ordered set of tiles. Finally, the profile sequence to traverse each tile is defined by mapping from the set of profiles within a tile to a temporally ordered set of segments $M_{U}\to M_{T}\;=\;\left\{1,\;\;\ldots ,\;\;M\right\}$ using an electrostatic repulsion criterion with periodic boundary conditions. Hence, a *distributed* temporal coverage is guaranteed both for the whole spectrum and within each tile. This strategy aids the aligned reconstruction conditioning by reducing the chances for large uncovered spectral areas due to head rotation. Figure 2C presents the Random‐checkered modification. Tiles are built as in the Checkered approach but segments are constructed by a random permutation of the tile elements drawn independently for each tile, so we have $m_{u_{e}}$. This guarantees a distributed coverage in probability and introduces some *incoherence* among profiles within and across segments. Figure 2D shows a Random view order where P is a random permutation of the profiles conforming the sequence. Note that, considering a free definition of segments, Sequential and Random schemes are particular cases of the Random‐checkered traversal respectively with tiling sizes of 1 × 1 and $K_{2}\;\times \;K_{3}$.

**Figure 3:**
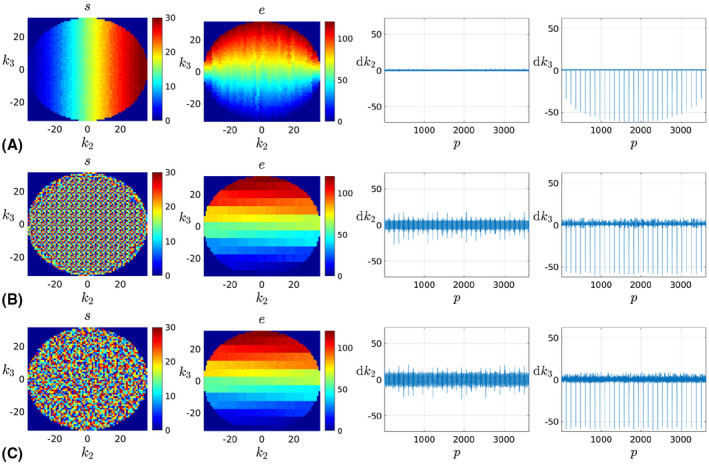
Segment *m* and tiling *e* temporal orders together with trajectory derivatives $dk_{2}$ and $dk_{3}$ (left to right) for Zig‐zag tiling orders used in non‐steady‐state sequences. The example corresponds to a case with *P* = 3630 profiles, *M* = 30 segments, and tiling pattern $U_{2}\;\times \;U_{3}\;=\;6\;\times \;5$. A, Sequential; B, Checkered; and C, Random‐checkered segments

**Figure 4:**
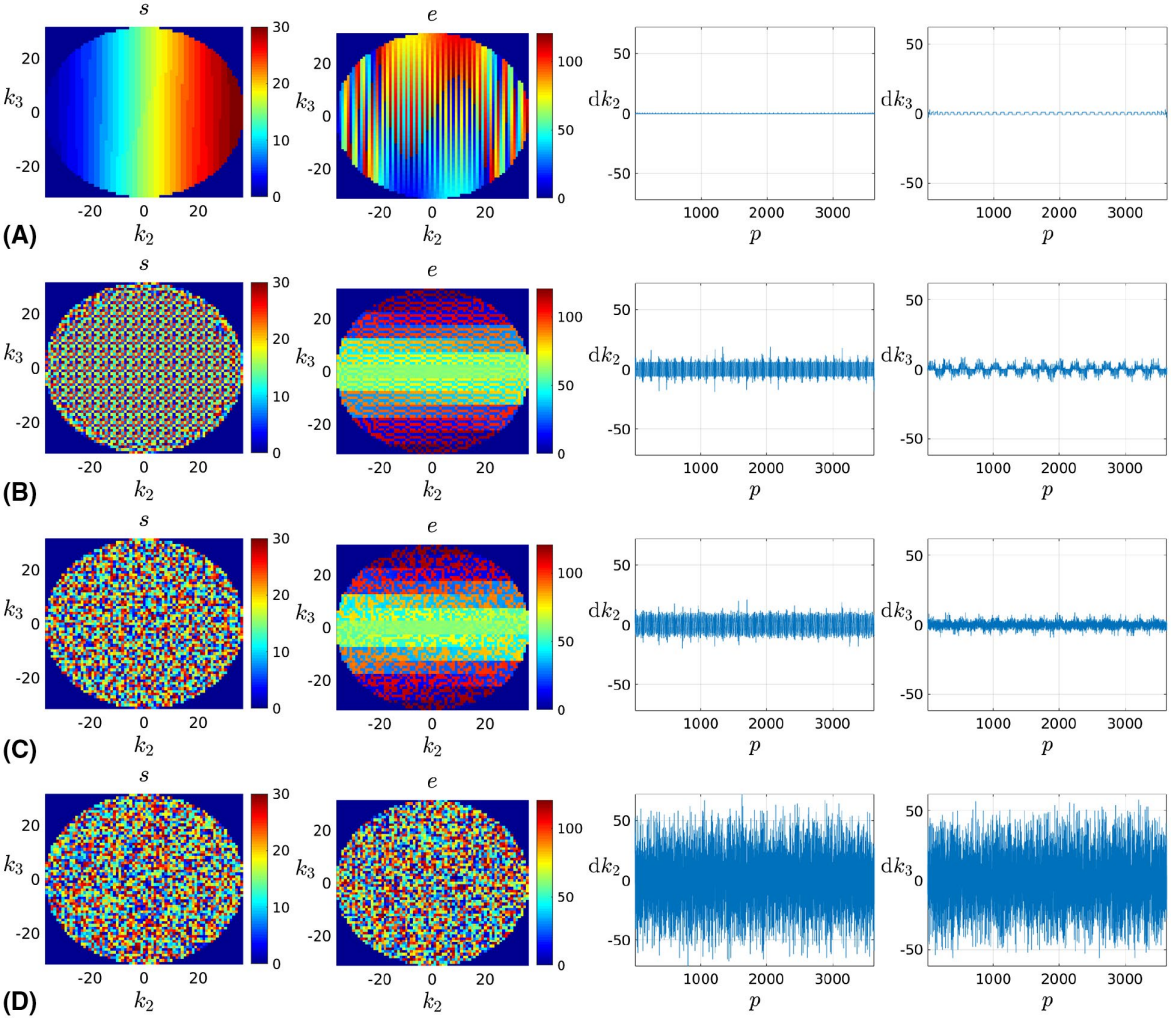
Segment *m* and tiling *e* temporal orders together with trajectory derivatives $dk_{2}$ and $dk_{3}$ (left to right) for Alternating zig‐zag tiling orders used in steady‐state sequences. The example corresponds to a case with *P* = 3630 profiles, *M* = 30 segments and tiling pattern $U_{2}\;\times \;U_{3}\;=\;6\;\times \;5$. A, Sequential; B, Checkered; C, Random‐checkered; and D, Random segments

View orders should preserve the contrast and the trajectory consistency. We establish the following differentiation:

1. **Non‐steady‐state sequences** (MP‐RAGE, FSE, FLAIR). They acquire a fraction of the k‐space or *shot* after each radiofrequency (RF) preparation. Thus, they induce a natural sampling partition where segments are in correspondence with shots. In addition, magnetic properties are not invariant for the different shot samples. Typically, middle samples within each shot cover the central area of the spectrum,^26^ which our orders can fulfill by jumping from tile to tile in a Zig‐zag manner. An example is shown in Figure 3 using an elliptical sampling area. The Checkered traversal produces regular segment patterns (first column), with neighboring colors maximally separated within the tile, whereas the Random‐checkered traversal produces non‐regular patterns. Tiling orders generate smooth color transitions across the spectrum for all presented traversals (second column), which translates into smooth magnetic properties of the profiles.
2. **Steady‐state sequences** (SPGR, bSSFP). They produce a temporally stable magnetization after reaching the steady‐state, usually facilitated by some preparatory dummy profiles, so the contrast becomes independent of the encoding order. For estimates attempted at the segment level, temporal resolvability of motion will increase with bigger tiling sizes $U_{2}U_{3}$. However, large jumps in the spectrum may induce inconsistencies due to eddy currents, especially for low repetition times, so a trade‐off may be required. If the profiles are covered by the application of *M* spectral *sweeps*, analogously to,^27^ eddy currents can be minimized by an Alternating zig‐zag tiling order where the traversal polarity is reversed for consecutive sweeps. This is illustrated in Figure 4. The segment structure (first column) matches that of Figure 3 but for some minor differences in the Sequential case due to smooth magnetization requirements in shot‐based sequences.^26^ The Sequential scheme guarantees a smooth passage through k‐space (third and fourth columns). Although quicker k‐space sweeps of our traversals imply larger $dk_{2}$ and $dk_{3}$ steps, these remain substantially lower than for the Random order, which should limit the impact of eddy currents. Finally, the Alternating zig‐zag suppresses the undesirable spikes in the fourth column of Figure 3.

### 2.3 Aligned reconstruction refinements

We propose a series of refinements for improved and more efficient aligned reconstructions:

1. **Spatial multiresolution**. The spatial and spectral grids for both subproblems in Equation 2 can be refined according to a given multiresolution pyramid as commonly used in image registration.^28^ In contrast to sequential sampling, the proposed orders allow for motion estimates from samples at coarse scales (area enclosed in cyan in Figure 2) to be completely exploited when reconstructing at fine scales. This is useful for quick aligned reconstructions as adequate motion estimates are often possible at coarse scales.
2. **Temporal multiresolution**. Motion estimation can also be attempted at intra‐shot (or intra‐sweep) levels (for instance using the samples enclosed within the yellow areas for the four intra‐segment subdivisions in Figure 2). However, estimates using low‐spatial harmonics localize the structures at coarse scales only and, conversely, motion estimation using high‐spatial harmonics alone is limited by lower SNR and prone to local optima. These limitations can be alleviated using hierarchical estimation refinements by temporally subdividing the samples considered by the motion states within a shot in a coarse‐to‐fine manner.
3. **Coil compression**. The two subproblems in Equation 2 can operate on a reduced number of virtual channels.^29^
4. **Motion compression**. The reconstruction subproblem complexity grows with the number of motion states, which can be reduced by motion compression or binning. Estimated motion parameter traces are approximated using piecewise constant functions by Haar wavelet decomposition truncation with threshold ***τ***. Thereby, original motion states are packed into effective states by grouping together those contiguous states with similar motion parameters into an effective motion parameter vector $\tilde{\theta }$. Thus, the reconstruction complexity is driven by the underlying motion complexity.
5. **Robustness**. Accurate intra‐shot corrections may be infeasible, for instance due to temporary inconsistencies in the magnetization. Denoting the real motion parameters by $\theta^{*}$, we can ideally characterize the loss $r_{x,\;\theta^{*}}$ using the sampling noise properties. Sampling noise follows a circularly symmetric complex Gaussian additive stationary distribution and, after whitening, it is independent across channels, so the losses per profile $r\left[m,\;e_{m}\right]\;=\;\sum_{c,k_{1}}r\left[m,\;e_{m},\;c,\;k_{1}\right]$ should ideally follow a $\chi^{2}$ distribution. To account for the sensitivity of the residuals to the underlying signal, we use trimmed statistics on a logarithmic scale $r_{b}\left[m\right]\;=\;P_{c(b)}^{E_{m}}\log\left(r\left[m,\;e_{m}\right]\right)$ with *b* ∈ 1, …*B* indexing the 100*c*(*b*) % centile $P_{c}$ of the loss distribution through k‐space $E_{m}$. As we are concerned with anomalously high residuals, robust estimates of the scale and mean of the statistic distribution across segments M are obtained respectively by $\sigma_{b}\;=\;\sqrt{2}\left(P_{c_{U}}^{M}r_{b}\left[m\right]-P_{c_{L}}^{M}r_{b}\left[m\right]\right)/\left({\text{erfc}}^{-1}\left(2c_{U}\right)-{\text{erfc}}^{-1}\left(2c_{L}\right)\right)$ and $\mu_{b}\;=\;P_{(c_{U}+c_{L})/2}^{M}r_{b}\left[m\right]+\sqrt{2}\sigma_{b}{\text{erfc}}^{-1}\left(c_{U}+c_{L}\right)$ choosing $c_{U}\;=\;0.25$ and $c_{L}\;=\;0.125$. Using these estimates, the statistics are normalized and averaged into $\bar{r}\left[m\right]\;=\;\sum_{b}\left(r_{b}\left[m\right]-\mu_{b}\right)/B\sigma_{b}$, and segments are weighted in the reconstruction by a matrix **W** with entries $w\left[m\right]\;=\;\min(M\;\text{erfc}\left(\bar{r}\left[m\right]/\sqrt{2}\right)/\left(2\tau_{w}\right),\;1)$, with $\tau_{w}$ an acceptance threshold corrected for multiple comparisons.
6. **Regularization**. If outlier segment rejection is activated or the reconstruction is applied to accelerated scans, some form of regularization may be advisable. This is considered by reformulating the reconstruction as:
  (3) $${\hat{x}}^{\left(i+1\right)}\;=\;\underset{x}{\text{argmin}}\|W^{1/2}\left(AFST_{{\tilde{\theta }}^{\left(i\right)}}x-y\right){\|}_{2}^{2}+2\lambda {\left\|Sx\right\|}_{1},$$
  where *λ* controls the degree of regularization and S corresponds to a shearlet decomposition, which provides nearly optimal approximation rates for piecewise smooth functions with discontinuities on a piecewise smooth surface.^30^ We resort to an iteratively reweighted least squares (IRWLS) solver, able to produce high‐quality solutions in a few iterations,^31^ with *λ* adaptively updated according to^32^ using a normalized Rayleigh‐quotient trace estimator.^33^

## 3 METHODS

### 3.1 Synthetic experiments

Our contributions are validated using a synthetic dataset built from a $T_{2}$ neonatal brain axial ground truth (GT) image without perceptible motion artifacts. This corresponds to a multi‐slice TSE sequence acquired on a 3 T philips achieva tx (same scanner as for in‐vivo tests) using a *C* = 32‐element neonatal head coil array, 0.8 × 0.8 mm in‐plane resolution, 1.6 mm slice thickness, echo time $T_{E}\;=\;145\;\text{ms}$, repetition time $T_{R}\;=\;12\;s$, and flip angle $\alpha \;=\;90^{\circ }$. Coil sensitivities were estimated from a separate reference scan.^34^ We use a 2D dataset and no regularization or outlier rejection for a concise presentation of results. We assume that the simulated 2D k‐space corresponds to the $k_{2}$‐$k_{3}$ PE plane of 3D scans and expect the driving conclusions to be extensible to 3D because estimates should be easier along the missing fully sampled readout direction.

Simulations were conducted to compare the conventional sequential order to the various proposed schemes as well as to characterize their performance. The forward model in the presence of rigid motion is applied to the GT to generate synthetically motion corrupted data. Synthesized measures are corrupted with noise levels corresponding to a mean SNR of 30 dB for reconstructions in the absence of motion or acceleration. Different degrees of motion are generated by drawing independent motion states uniformly at random on an interval of rotations [−*θ*/2, *θ*/2] around the field of view (FOV) center. Satisfactory convergence in the presence of noise can be ascertained on the assumption of an identifiable global optimum basin by $r_{\hat{x},\;\hat{\theta }}\leq r_{\hat{x},\;\theta^{*}}$. In this case, the error in the motion parameters $\hat{\theta }-\theta^{*}$ is attributed to the uncertainty from the measurement noise and not to partial convergence. Note that we can generally achieve a lower loss for the joint problem ($r_{\hat{x},\;\hat{\theta }}$) than with the knowledge of the motion parameters ($r_{\hat{x},\;\theta^{*}}$) due to the larger complexity of the former. Reconstructions are terminated when $r_{\hat{x},\;\hat{\theta }}\leq r_{\hat{x},\;\theta^{*}}$ and the abscissa scale of the convergence plots was chosen so that iterations have a direct translation into computational costs.

### 3.2 In vivo experiments

In vivo experiments include the main families of volumetric sequences for brain MRI (see Table 1). We have performed a controlled motion experiment on a consented adult volunteer and applied the method to replace sedation on pediatric subjects scanned after written informed parental consent for an epilepsy study. Imaging is performed using a 32‐channel adult head coil. Data are acquired in the inferior‐superior (IS) $k_{1}$, anterior‐posterior (AP) $k_{2}$ and left‐right (LR) $k_{3}$ orientation using our scanner implementation of the Random‐checkered traversal. This way, potentially strongest rotations on the sagittal plane are captured by the $k_{1}$‐$k_{2}$ coordinates, which may increase the resolvability of intra‐shot motion. In addition, IS readouts allow to easily downweight additional motion sources within the FOV when estimating for motion by restricting the loss function to the superior part of the FOV (2/3 in our implementation). Finally, this orientation facilitates nonselective RF excitation pulses for shorter $T_{R}$.

**Table 1:** Sequence parameters for the different modalities considered in the experiments

Subjects	Modality	$T_{R}$	$T_{E}$	$T_{I}$	*α*	*P*	$U_{2}\;\times \;U_{3}$	$\Delta_{y}$	*R*	${\text{FOV}}_{x}$	*t*
Adult	MP‐RAGE	8.3 ms	3.7 ms	1.00 s	$8.0^{\circ }$	3630	6 × 5	1.5 mm	2 × 2	240 × 240 × 220 mm	1 min 29 s
Pediatric	MP‐RAGE	7.0 ms	2.3 ms	0.90 s	$8.0^{\circ }$	17920	8 × 10	1.0 mm	1.4 × 1.4	240 × 240 × 188 mm	3 min 07 s
	TSE	2.50 s	0.31 s	–	$90.0^{\circ }$	17955	9 × 15				5 min 42 s
	FLAIR	5.00 s	0.41 s	1.80 s		18200	10 × 10				8 min 30 s
	SPGR	8.2 ms	3.0 ms	–	$12.3^{\circ }$	21654			1.3 × 1.3		3 min 06 s
	bSSFP	6.0 ms	3.0 ms		$46.0^{\circ }$						2 min 16 s

*Note*: $T_{I}$ is the inversion time, $\Delta_{y}$ the acquired resolution, *R* the uniform acceleration factor, ${\text{FOV}}_{x}$ the reconstructed FOV, and *t* the scan duration.

In the controlled motion experiment, the volunteer was first scanned without deliberate motion, and then asked to perform extreme and continuous motion for the entire scan, which was repeated three times. Reconstructed volumes are jointly registered together for error comparisons. The pediatric cohort includes 26 subjects ranging from 3 to 19 years old (mean ± SD of 12 ± 5 years), typically acquiring one MP‐RAGE, TSE, and FLAIR, two SPGRs, and three bSSFPs for an approximate total of 208 tested volumes across all participants. Strongest artifacts in our data are generally arising from motion, so the reported case has been separately chosen for each modality as the most artifacted after reconstruction without motion correction.

### 3.3 Implementation details

In the in vivo experiments sensitivities are compressed into a number of channels corresponding to a 10% SNR loss. The number of resolution levels is defined as $L\;=\;\left\lfloor \log_{2}\left(4\;\text{mm}/\Delta_{y}^{\min}\right)\right\rfloor +1$, with ⌊·⌋ denoting the biggest integer lower or equal than the argument and $\Delta_{y}^{\min}$ the minimum of the voxel sizes along different directions. As we use 2 × subsampling ratios, we operate at a minimum resolution of 4 mm. In the first iteration at level *l*, a soft‐masked^35^ full CG reconstruction is run till loss reduction saturation. Then, the method quickly alternates between reconstruction and motion correction using one CG and one LM iteration with heuristically updated damping and line search. We activate a flag for provisional convergence of the parameters of a given motion state when the maximum update is smaller than a threshold $\tau_{\Delta \theta }\;=\;\left\{0.05,\;0.02^{\circ }{\text{mm}}^{-1}\right\}\Delta_{y^{l}}^{\max},$ with same values used for motion compression. This saves computations by considering motion updates only on not‐converged parameters. However, this flag is reset to 0 whenever *i* = *n*(*n*−1)/2+1 ($n\in N_{>\;0}$) to account for the impact of the updated reconstructions in the motion parameter estimates. Joint convergence is achieved when provisional convergence is achieved for all motion states. Then, the method runs a full CG reconstruction with the consolidated motion parameters. If regularized outlier rejection reconstructions are activated, artifacted segments are rejected at levels *l* such that $\Delta_{y^{l}}^{\max}\leq 2\;\text{mm}$ by densely sampling within $\left[c\left(1\right),\;c\left(B\right)\right]\;=\;0.5+\left(0.35/\max\left(\Delta_{y^{l}}^{\max},\;1\right)\right)\left[-1,\;1\right]$ and using $\tau_{w}\;=\;0.05$. If regularization is applied, shearlets are designed based on^36^ and a final reconstruction is launched with 3 CG iterations within 2 updates of the IRWLS‐induced cost function and *λ*. Reconstructions are performed on a 8(16) × intel(r) core(tm) i7‐5960x 3.00 GHz CPU, 64 GB RAM, geforce gtx titan x GPU. For further implementation details, readers can refer to the source code.

## 4 RESULTS

### 4.1 Validation

**Figure 5:**
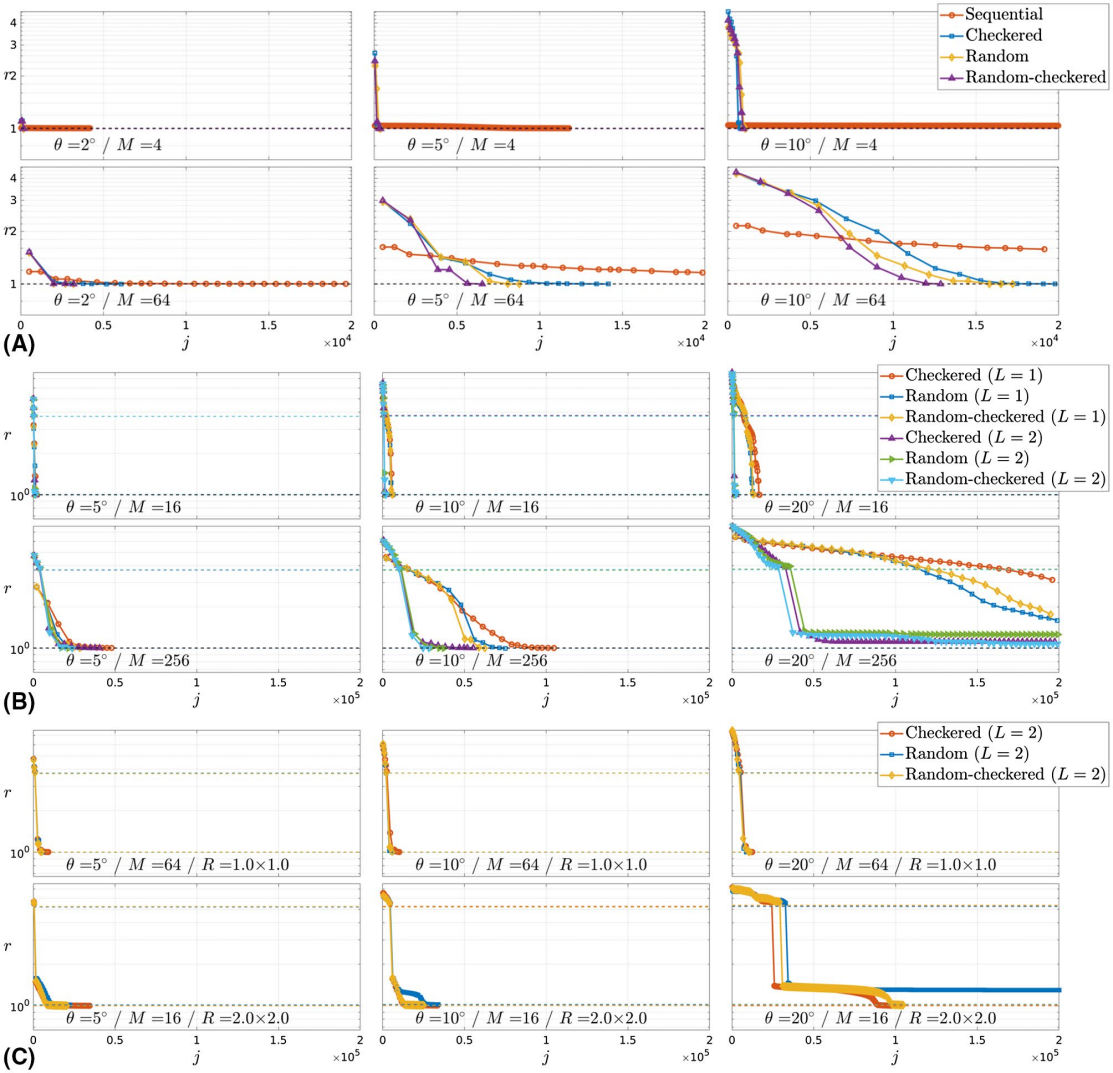
Aligned reconstruction convergence against effective iterations *j* defined as a single application of the encoding or decoding operator for a single motion state and coil channel at full resolution. A, Different encoding orders, number of segments *M* ∈ {4, 64} (rows), motion levels $\theta \in \{2^{\circ },\;5^{\circ },\;10^{\circ }\}$ (columns), $j_{\max}\;=\;20000$. B, Different encoding orders and number of multiresolution levels *L*, number of segments *M* ∈ {16, 256} (rows), motion levels $\theta \in \{5^{\circ },\;10^{\circ },\;20^{\circ }\}$ (columns), $j_{\max}\;=\;200000$. C, Different encoding orders, *L* = 2, acceleration factors *R* ∈ {1 × 1,2 × 2} with matched number of segments *M* ∈ {64, 16} (rows), motion levels $\theta \in \{5^{\circ },\;10^{\circ },\;20^{\circ }\}$ (columns), $j_{\max}\;=\;200000$

**Figure 6:**
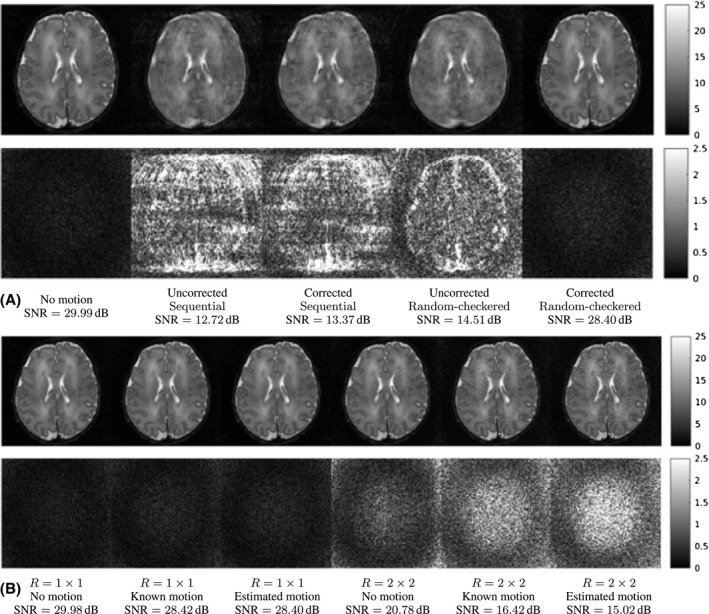
Reconstruction results (A) without and with motion correction for Sequential and Random‐checkered traversals compared to the GT (top row) and corresponding error maps (bottom row) for *M* = 64/$\theta \;=\;10^{\circ }$ and (B) for a Random‐checkered traversal without motion, and with known and estimated $\theta \;=\;10^{\circ }$ motion for *R* = 1 × 1 and *R* = 2 × 2 (top row) and corresponding error maps (bottom row)

In Figure 5, we compare different simulated reconstruction scenarios showing the losses when iterating the method, $r_{{\hat{x}}^{\left(i+1\right)},\;{\hat{\theta }}^{\left(i\right)}}$ as solid‐colored lines with joint iterations represented by markers. Losses in the convergence plots are normalized to the minimum of the reference levels $r_{\hat{x},\;\theta^{*}}$, which are shown as dashed lines strongly overlapped for the different alternatives. Figure 6 includes reconstructions with and without motion correction for different reconstruction scenarios and provides absolute value error maps and mean SNR for the compared cases.

#### 4.1.1 Encoding orders

Figure 5A compares the Sequential, Checkered, Random‐checkered, and Random traversals. Global convergence is achieved for all considered *M* and *θ* when using any of the Checkered, Random‐checkered, or Random traversals. In contrast, when using the Sequential order, the method converges to a local optimum or fails to converge in the prescribed iterations except for $\theta \in \{2^{\circ },\;5^{\circ }\}$/*M* = 4. The loss at the first iteration $r_{{\hat{x}}^{\left(1\right)},\;{\hat{\theta }}^{\left(0\right)}}$ is always bigger when using nonsequential traversals. This increased inconsistency in the measurement domain relates to the aligned reconstruction sensitivity to motion degradation.

Figure 6A shows reconstructions and errors with and without motion correction for the Sequential and Random‐checkered traversals together with GT motion‐free reconstructions. Motion‐corrected reconstructions using the Random‐checkered data appear similar to the GT despite the strong blurring in uncorrected reconstructions. This is confirmed by the lack of perceptible structure in the residuals and a moderate noise amplification. In contrast, corrections using the Sequential traversal provide only a modest visual benefit.

#### 4.1.2 Multiresolution

Figure 5B compares the Checkered, Random‐checkered, and Random traversals when using a single scale for joint motion estimation and reconstruction (*L* = 1) and when first approximating the motion solution at half the acquired resolution to initialize the joint problem at full resolution (*L* = 2). The Sequential traversal was excluded because, as discussed when introducing the multiresolution strategy, it has no opportunity to improve from the poor relative performance showed in Figure 5A by exploiting multiresolution. Plots also include $r_{\hat{x},\;\theta^{*}}$ at the coarse scale. Global convergence is achieved for all traversals at all considered configurations except at *M* = 256/$\theta \;=\;20^{\circ }$. However, the multiresolution strategy (*L* = 2) achieves global convergence in less iterations or provides a solution with lower residuals (*M* = 256/$\theta \;=\;20^{\circ }$). For moderate levels of motion, convergence is generally quick. For instance, it takes approximately 10 joint iterations *i* when using the Random‐checkered traversal in a case where random excursions of up to $\theta \;=\;10^{\circ }$ are imposed in every one of the *M* = 256 segments, probably a more challenging scenario than expected in practice.

#### 4.1.3 Acceleration

Figure 5C tests the ability of the Checkered, Random‐checkered, and Random traversals (using *L* = 2 scales) to operate in uniformly accelerated regimes as given by different acceleration factors *R*. We observe convergence to the global solution in all tested scenarios aside from the Random traversal at *R* = 2 × 2/*M* = 16/$\theta \;=\;20^{\circ }$. Considering all conducted simulations, the random checkered traversal is generally providing the quickest solutions.

Figure 6B provides an example of reconstructions and errors in the absence of motion, with known motion and with estimated motion at *R* = 1 × 1 and *R* = 2 × 2. The SNR figures for *R* = 1 × 1 and known motion show a degradation of 1.56 dB with respect to the reference due to noise amplification from nonuniform effective k‐space sampling after motion. No further degradation is introduced from motion estimation errors, as approximately the same SNR figures are obtained for known and estimated motion. *R* = 2 × 2 acceleration in the absence of motion introduces a degradation of 9.20 dB with respect to the reference, which stems from the reduced number of samples and the g‐factor.^37^ The presence of motion adds further degradations quantified as 4.36 dB, thus stronger than in the non‐accelerated case. Therefore, without regularization, the limiting reconstruction quality in the presence of motion decreases with larger distances between neighboring k‐space points. Finally, accelerating the scan has also an impact in the uncertainty of motion estimates, as we observe a degradation of 1.40 dB from known to estimated motion, although the errors show no perceptible structure.

### 4.2 Redundancy for motion tolerance

**Figure 7:**
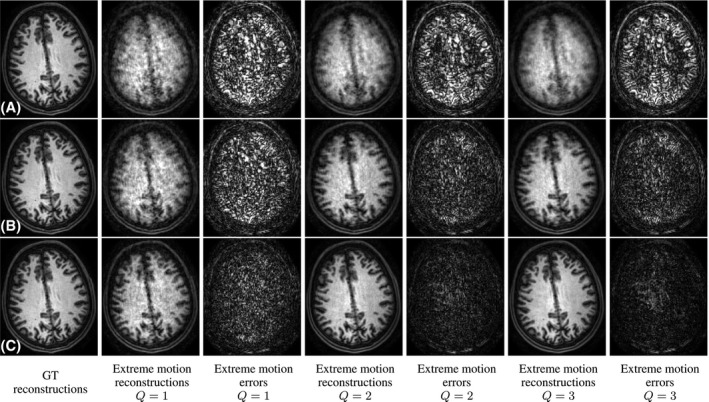
Reconstruction results for extreme motion in vivo. A, Uncorrected; B, inter‐shot corrections; and C, intra‐shot corrections. From left to right, results without deliberate motion and reconstructions and errors in the presence of extreme motion for *Q* = {1, 2, 3} repeats of a *R* = 2 × 2 accelerated baseline scan

Figure 7 compares reconstructions without correction, with inter‐shot corrections and when activating intra‐shot corrections in the presence of extreme motion during in vivo data acquisition. Intra‐shot corrections are triggered by subsequent temporal binary subdivisions of the sampled information within each shot until 16 motion states are estimated per shot. We show reconstructions without deliberate motion (GT reconstructions), and reconstructions and absolute differences with respect to the GT using *Q* = 1, *Q* = 2, and *Q* = 3 repeats under extreme motion (Extreme motion reconstructions/errors, *Q* = {1, 2, 3}). Results for *Q* = 1 and *Q* = 2 correspond to the first repeats, with no remarkable differences observed when choosing any other combination. Reconstructions are provided without regularization or outlier rejection. Results without deliberate motion show that inter‐ and intra‐shot corrections do not reduce the reconstruction quality, which demonstrates a safe application of generalized reconstructions in the absence of motion. Degradation is noticeable for uncorrected reconstructions in the presence of motion for all values of *Q*, although with less coherent ghosts as *Q* increases due to incoherent blurring by Random‐checkered motion averaging. Inter‐shot corrections increase the reconstruction quality in all cases, with more finely resolved cortical structures as *Q* increases but with noticeably inferior quality than without deliberate motion. Residual degradation is only partially accounted when using intra‐shot corrections on a single repeat, but can be more satisfactorily addressed with *Q* = 2 and even more with *Q* = 3. Namely, the level of deblurring in the fourth and sixth columns of Figure 7C makes corresponding reconstructions visually comparable to those of the first column despite the extreme and continuous motion (estimated excursions up to $25^{\circ }$). Thus, we can reason that powerful tolerance is achieved for *R* = 2 × 2 and *Q* = 2, so that *Q* = 1 with acceleration $R\;=\;\sqrt{2}\;\times \;\sqrt{2}$ (Both alternatives involve the same scanning time but the latter generates a lower g‐factor. The former was used in this experiment because it was more convenient to our scanner implementation of the traversals.) may be adequate for motion tolerance in practice, which has been used to guide the acceleration in the pediatric cohort (see Table 1). However, in contrast to computation times of 2 min (nondeliberate motion), and 11 min (extreme motion, *Q* = 3) for inter‐shot corrections, corresponding intra‐shot corrections required 21 min and 20 h 36 min. Thus, despite being technically feasible, intra‐shot corrections may have limited applicability due to computational costs. Computational cost increase with the complexity of motion is due to the larger number of iterations for convergence and to the proposed motion compression strategy, with 13/30 binned inter‐shot motion states without deliberate motion and 30/30 with extreme motion (*Q* = 1), with proportional savings in the reconstruction steps in the former.

### 4.3 Noncompliant subjects

**Figure 8:**
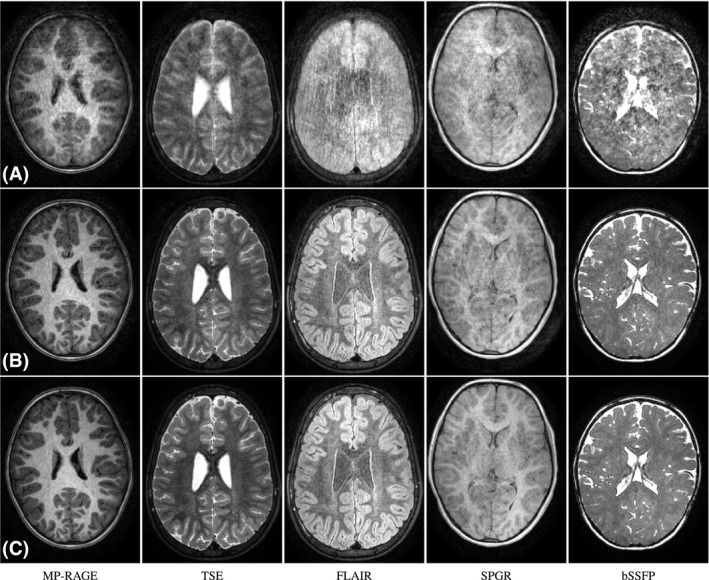
Reconstruction results for pediatric cases with largest intra‐scan degradations. A, Uncorrected; B, motion‐corrected; and C, motion‐corrected and regularized outlier segment rejection reconstructions. From left to right, results for the main families of sequences for structural brain imaging

Figure 8 shows worst‐case reconstructions without motion correction, with motion correction alone and with motion correction and the regularized outlier segment rejection. Results are shown for main structural brain MRI modalities, MP‐RAGE, TSE, FLAIR, SPGR, and bSSFP. In all sequences we observe a substantial improvement when activating motion‐corrected reconstructions alone, with better delineated cortical structures. However, subtle artifacts are still present, either in the form of ghosts or of coloured noise. Figure 8C shows that quality can be further improved by rejecting the less consistent segments and performing a regularized reconstruction. In some sequences discarding the artifacted segments seems to reduce residual artifacts from uncorrected fast motion (see for instance fine details in SPGR) while in others it seems to mainly improve the magnetization consistency (see TSE contrast). Across the cohort, we have observed that motion artifact levels always decrease when compensating for motion, with no remarkable differences when activating the corrections in the absence of artifacts. This is along the lines of the quantitative population metrics obtained for the less favourable sequential sampling^20^ or for multi‐slice scans.^38^ Worst‐case results of Figure 8 have been judged satisfactory by the practitioners and researchers involved in the project. Therefore, the proposed methodology is delivering reliable examinations for unsedated pediatric subjects challenging to comply to the MRI motion requirements. In this experiment, motion estimates were performed at half the acquired resolution with joint motion estimation and reconstruction always taking less time than final reconstructions at full resolution. Computation times range between 5 min in least artifacted and 40 min in most artifacted volumes in our cohort. Estimated motion traces and outlier segments are reported in Supporting Information Figure S1.

## 5 DISCUSSION

We have presented DISORDER, a retrospective framework for motion‐tolerant structural 3D k‐space encoded brain imaging that combines optimized view orders with an improved aligned reconstruction. The proposed distributed and incoherent orders increase the motion sensitivity of the information sampled within a given time window which, provided a certain degree of redundancy, enables the resolvability of motion in the reconstruction. Conducted simulations have shown that reordering the k‐space traversals introduces a significant boost in the ability to estimate the head pose and suppress motion artifacts. Tolerance to motion has been demonstrated in vivo on a controlled experiment involving extreme and continuous motion throughout the examination as well as for the main families of sequences used for structural brain imaging by presenting the reconstruction results on the most challenging datasets from a pediatric cohort of 26 subjects.

Although DISORDER is robust enough in its current form so as to be of practical interest for reliable structural brain MR examinations in noncompliant cohorts, with plans in our center to use it to progressively replace unnecessary sedation or anesthesia in pediatric and neonatal populations,^39^ it is obviously not free from limitations. First, data consistency may be affected by additional degrading factors. These include inaccuracies in sensitivities but also water‐fat shifts, eddy currents, or flow artifacts. In practice, applying fat suppression when possible, designing the tiles for adequate trade‐offs between eddy currents and motion resolvability in bSSFP sequences, and adequate planning and scanning procedures are usually sufficient to address these issues. Differently, correction of non‐rigid motion components would require an extension of the formulation. Although analogous methodologies^40^ have shown potential in this context, a robust and efficient extension to non‐rigid motion models will probably require a careful computational design. This may be particularly the case for high‐resolution applications, where both rigid and non‐rigid motion become more important and challenging to correct.^2^ Moreover, coarse scale motion at ultra high field may require additional corrections of high‐order effects. Finally, in this manuscript we have restricted ourselves to uniform sampling, with further work required to generalize the incoherent and distributed orders and characterize motion correction and resolution retrieval when using variable densities.

In the in vivo experiments of Figure 8, we have shown that inter‐shot corrections can be sufficient in practical brain imaging scenarios requiring motion tolerance. Our underlying assumption is that the subject remains approximately still for a significant portion of the acquisition. In this case, inter‐shot corrections are enough to reconcile the brain pose among the stable periods and data rejection can be applied to the transitions, again, provided that sampling is redundant enough. However, intra‐shot corrections may become more important in challenging situations, as illustrated in Figure 7. Despite its computational limitations, our method is able to provide stable intra‐shot estimates in the absence of motion while offering certain motion correction potential. Although a prior model for the temporal evolution of motion may aid in certain applications, in general, limitations arising from available computational resources and SNR per motion state are likely to complicate intra‐shot tractability.

The situation may perhaps be different if using supervised learning strategies to inform the exploration of the motion parameter space. These may help to improve the spatiotemporal resolvability of motion by aiding the intra‐shot corrections to find better motion solutions. Training may also help to enlarge the motion capture range at a given level of redundancy or decrease the required level of redundancy for a given motion capture range. Although direct learning of motion‐corrected reconstructions could also be attempted, it is likely that, in many circumstances, better results will be obtained when concatenating learned reconstructions with model‐based strategies, as recently suggested in.^41^ Further integration of both approaches could be tackled, for instance, by incorporating the motion operator into the model‐based learning framework in,^42^ which may be effective in dealing with the residual penalties from g‐factor amplification due to motion (see Figure 6B). Thereby, future work will explore the opportunities for extending the ranges of motion resilience by supervised learning.

## 6 CONCLUSION

We have proposed a simple modification of standard 3D Cartesian sequences for structural brain imaging, involving only a distributed and incoherent reordering of the sampled profiles, for high‐quality imaging in the presence of motion. Improved convergence has been demonstrated when using a separable nonlinear least squares formulation for joint motion estimation and reconstruction. Feasibility and conditions for inter‐ and intra‐shot corrections have been characterized by simulations and in vivo reconstructions under extreme motion. The DISORDER method has been successfully applied to replace sedation in a pediatric population scanned using common clinical examination protocols by combining inter‐shot corrections with regularized outlier segment rejection reconstructions. Future work will focus on applying DISORDER to other cohorts and on strengthening its performance by integrating motion learning strategies.

## Supporting information


**FIGURE S1** First 120 s of estimated motion traces for pediatric cases with largest intra‐scan degradations for each sequence. Left: original motion traces. Right: motion traces with segment opacity given by corresponding reliability *w*. Solid lines indicate data collection periods for each segment with dotted lines used to connect these
