# Supplementary material for: Motion‐corrected MRI with DISORDER: Distributed and incoherent sample orders for reconstruction deblurring using encoding redundancy
Source: Magn Reson Med. 2020 Jan 3;84(2):713–26. doi: 10.1002/mrm.28157 (PMC7392051; doi:10.1002/mrm.28157)
Supplement: Supplementary file 1 — FIGURE S1 First 120 s of estimated motion traces for pediatric cases with largest intra‐scan degradations for each sequence. Left: original motion traces. Right: motion traces with segment opacity given by corresponding reliability w. Solid lines indicate data collection periods for each segment with dotted lines used to connect these [file MRM-84-713-s001.pdf]

SUPPORTING INFORMATION

Fig. S1 collects the estimated motion traces for the cases in Fig. 8. Although no temporal regularization is used, all traces show periods of stability, which suggests accurate estimates at least in these periods. In Fig. S1 the opacity of the traces is driven by the segment weights from the proposed outlier detection method. We observe that outliers generally correspond to main motion transients, in agreement to higher chances for intra-sweep degradation in these periods.

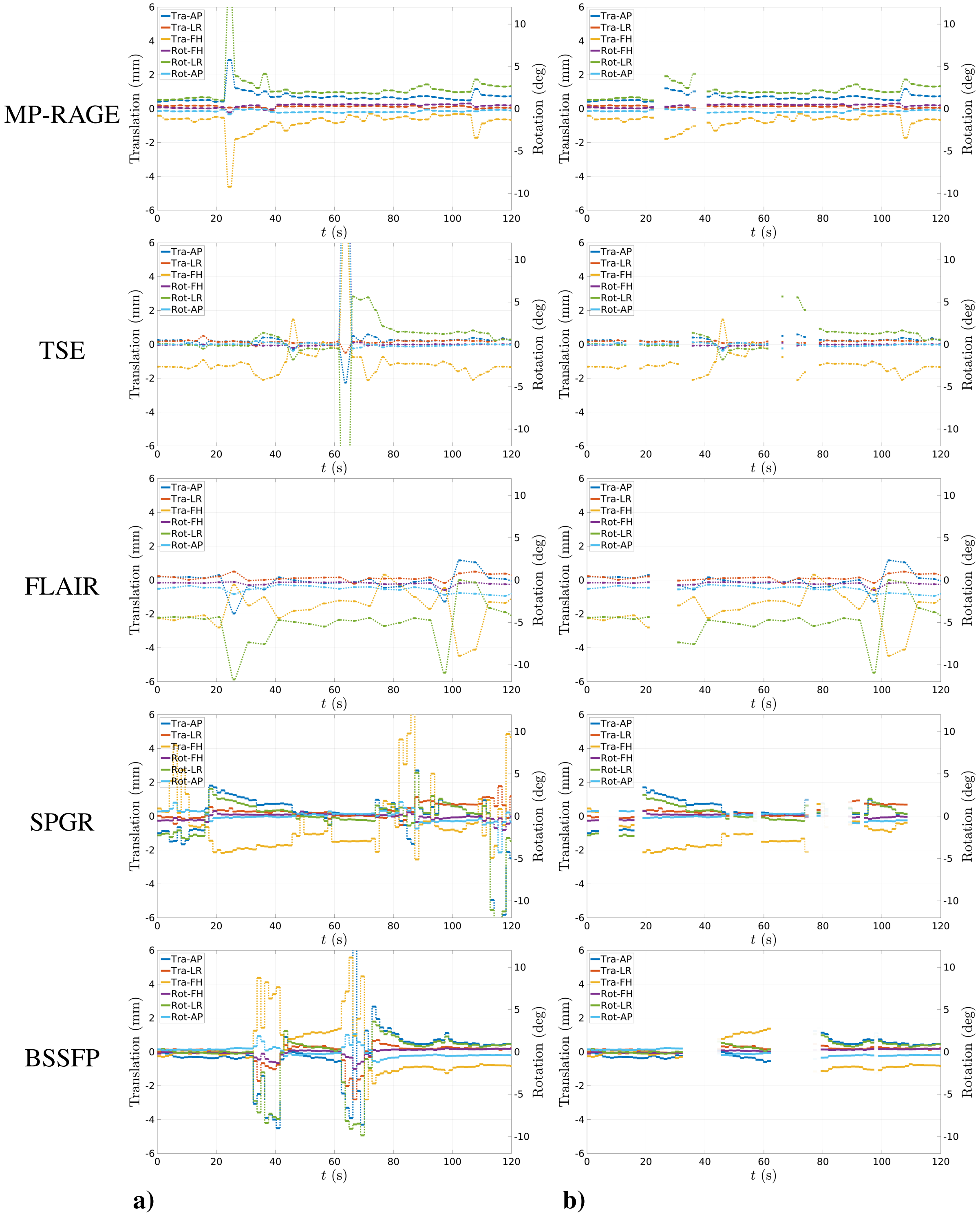

Figure S1. First 120 s of estimated motion traces for pediatric cases with largest intra-scan degradations for each sequence. Left: original motion traces. Right: motion traces with segment opacity given by corresponding reliability w. Solid lines indicate data collection periods for each segment with dotted lines used to connect these.
